# Supplementary material for: Antimicrobial activity of Tachyplesin 1 against Burkholderia pseudomallei: an in vitro and in silico approach
Source: PeerJ. 2016 Oct 25;4:e2468. doi: 10.7717/peerj.2468 (PMC5088614; doi:10.7717/peerj.2468)
Supplement: Supplemental Information 1 [file peerj-04-2468-s001.docx]

Supplementary 1

List of strains used in the preliminary study

All strains are *B. pseudomallei* unless stated (S- susceptible, R- resistant)

|  | | | **Published** | **Published** | **Study** | Magainin 2 | Sushi 1 | Sushi 2 | Ornithine | V2D | 1037 | 1018 | DJK 5 |
| --- | --- | --- | --- | --- | --- | --- | --- | --- | --- | --- | --- | --- | --- |
| **Type of strains** | **Source** | **Label** | LL-37 | PG1 | TP1 |  |  |  |  |  |  |  |  |
| Clinical |  | UMC002 | S | S | S | R | R | R | R | R | R | R | R |
| Clinical |  | UMC006 | S | S | S | R | R | R | R | R | R | R | R |
| Clinical |  | UMC007 | S | S | S | R | R | R | R | R | R | R | R |
| Clinical |  | UMC009 | S | S | S | R | R | R | R | R | R | R | R |
| Clinical |  | UMC010 | S | S | S | R | R | R | R | R | R | R | R |
| Clinical |  | UMC011 | S | S | R | R | R | R | R | R | R | R | R |
| Clinical |  | UMC012 | S | S | S | R | R | R | R | R | R | R | R |
| Clinical |  | UMC013 | S | S | S | R | R | R | R | R | R | R | R |
| Clinical |  | UMC014 | S | S | S | R | R | R | R | R | R | R | R |
| Clinical |  | UMC015 | S | S | S | R | R | R | R | R | R | R | R |
| Clinical |  | UMC016 | S | S | S | R | R | R | R | R | R | R | R |
| Clinical |  | UMC019 | S | S | S | R | R | R | R | R | R | R | R |
| Clinical |  | UMC020 | S | S | R | R | R | R | R | R | R | R | R |
| Clinical |  | UMC021 | S | S | S | R | R | R | R | R | R | R | R |
| Clinical |  | UMC022 | S | S | S | R | R | R | R | R | R | R | R |
| Clinical |  | UMC023 | S | S | S | R | R | R | R | R | R | R | R |
| Clinical |  | UMC024 | S | S | S | R | R | R | R | R | R | R | R |
| Clinical |  | UMC025 | S | S | S | R | R | R | R | R | R | R | R |
| Clinical |  | UMC026 | S | S | S | R | R | R | R | R | R | R | R |
| Clinical |  | UMC027 | S | S | S | R | R | R | R | R | R | R | R |
| Clinical |  | UMC028 | S | S | R | R | R | R | R | R | R | R | R |
| Clinical |  | UMC029 | S | S | S | R | R | R | R | R | R | R | R |
| Clinical |  | UMC030 | S | S | S | R | R | R | R | R | R | R | R |
| Clinical |  | UMC031 | S | S | S | R | R | R | R | R | R | R | R |
| Clinical |  | UMC032 (K96243) | S | S | S | R | R | R | R | R | R | R | R |
| Clinical |  | UMC033 | S | S | S | R | R | R | R | R | R | R | R |
| Clinical |  | UMC034 | S | S | R | R | R | R | R | R | R | R | R |
| Clinical |  | UMC035 | S | S | S | R | R | R | R | R | R | R | R |
| Clinical |  | UMC036 | S | S | S | R | R | R | R | R | R | R | R |
| Clinical |  | UMC038 | S | S | R | R | R | R | R | R | R | R | R |
| Clinical |  | UMC039 | S | S | S | R | R | R | R | R | R | R | R |
| Clinical |  | UMC040 | S | S | S | R | R | R | R | R | R | R | R |
| Clinical |  | UMC042 | S | S | S | R | R | R | R | R | R | R | R |
| Clinical |  | UMC043 | S | S | S | R | R | R | R | R | R | R | R |
| Clinical |  | UMC044 | S | S | S | R | R | R | R | R | R | R | R |
| Clinical |  | UMC045 | S | S | S | R | R | R | R | R | R | R | R |
| Clinical |  | UMC046 | S | S | S | R | R | R | R | R | R | R | R |
| Clinical |  | UMC047 | S | S | S | R | R | R | R | R | R | R | R |
| Clinical |  | UMC048 | S | S | R | R | R | R | R | R | R | R | R |
| Clinical |  | UMC049 | S | S | S | R | R | R | R | R | R | R | R |
| Clinical |  | UMC049L | S | S | S | R | R | R | R | R | R | R | R |
| Clinical |  | UMC049S | S | S | R | R | R | R | R | R | R | R | R |
| Clinical |  | UMC050 | S | S | S | R | R | R | R | R | R | R | R |
| Clinical |  | UMC051 | S | S | S | R | R | R | R | R | R | R | R |
| Clinical |  | UMC052 | S | S | S | R | R | R | R | R | R | R | R |
| Clinical |  | UMC053 | S | S | R | R | R | R | R | R | R | R | R |
| Clinical |  | UMC054A | S | S | S | R | R | R | R | R | R | R | R |
| Clinical |  | UMC054B | S | S | S | R | R | R | R | R | R | R | R |
| Clinical |  | UMC055 | S | S | S | R | R | R | R | R | R | R | R |
| Clinical |  | UMC056 | S | S | R | R | R | R | R | R | R | R | R |
| Clinical |  | UMC057 | S | S | S | R | R | R | R | R | R | R | R |
| Clinical |  | UMC058 | S | S | S | R | R | R | R | R | R | R | R |
| Clinical |  | UMC059 | S | S | S | R | R | R | R | R | R | R | R |
| Clinical |  | UMC061 | S | S | R | R | R | R | R | R | R | R | R |
| Clinical |  | UMC061A | S | S | S | R | R | R | R | R | R | R | R |
| Clinical |  | UMC061B | S | S | S | R | R | R | R | R | R | R | R |
| Clinical |  | UMC062 | S | S | R | R | R | R | R | R | R | R | R |
| Clinical |  | UMC063 | S | S | S | R | R | R | R | R | R | R | R |
| Clinical |  | UMC064 | S | S | S | R | R | R | R | R | R | R | R |
| Clinical |  | UMC065 | S | S | S | R | R | R | R | R | R | R | R |
| Clinical |  | UMC066 | S | S | S | R | R | R | R | R | R | R | R |
| Clinical |  | UMC067 | S | S | S | R | R | R | R | R | R | R | R |
| Clinical |  | UMC068 | S | S | S | R | R | R | R | R | R | R | R |
| Clinical |  | UMC069 | S | S | R | R | R | R | R | R | R | R | R |
| Clinical |  | UMC070 | S | S | S | R | R | R | R | R | R | R | R |
| Clinical |  | UMC071 | S | S | S | R | R | R | R | R | R | R | R |
| Clinical |  | UMC072 | S | S | S | R | R | R | R | R | R | R | R |
| Clinical |  | UMC073 | S | S | R | R | R | R | R | R | R | R | R |
| Clinical |  | UMC074 | S | S | S | R | R | R | R | R | R | R | R |
| Clinical |  | UMC074L | S | S | S | R | R | R | R | R | R | R | R |
| Clinical |  | UMC074S | S | S | S | R | R | R | R | R | R | R | R |
| Clinical |  | UMC076 | S | S | S | R | R | R | R | R | R | R | R |
| Clinical |  | UMC077 | S | S | R | R | R | R | R | R | R | R | R |
| Clinical |  | UMC078 | S | S | S | R | R | R | R | R | R | R | R |
| Clinical |  | UMC079 | S | S | S | R | R | R | R | R | R | R | R |
| Clinical |  | UMC080 | S | S | S | R | R | R | R | R | R | R | R |
| Clinical |  | UMC081 | S | S | S | R | R | R | R | R | R | R | R |
| Clinical |  | UMC082 | S | S | S | R | R | R | R | R | R | R | R |
| Clinical |  | UMC084 | S | S | R | R | R | R | R | R | R | R | R |
| Clinical |  | UMC085 | S | S | S | R | R | R | R | R | R | R | R |
| Clinical |  | UMC086 | S | S | S | R | R | R | R | R | R | R | R |
| Clinical |  | UMC087 | S | S | S | R | R | R | R | R | R | R | R |
| Clinical |  | UMC088 | S | S | R | R | R | R | R | R | R | R | R |
| Clinical |  | UMC089 | S | S | S | R | R | R | R | R | R | R | R |
| Clinical |  | UMC105 | S | S | R | R | R | R | R | R | R | R | R |
| Clinical |  | UMC106 | S | S | S | R | R | R | R | R | R | R | R |
| Clinical |  | UMC107 | S | S | S | R | R | R | R | R | R | R | R |
| Clinical |  | UMC108 | S | S | S | R | R | R | R | R | R | R | R |
| Clinical |  | UMC109 | S | S | S | R | R | R | R | R | R | R | R |
|  |  | | | | | | | | | | | | |
| Animal |  | UMA001 | S | S | S | R | R | R | R | R | R | R | R |
| Animal |  | UMA002 | S | S | S | R | R | R | R | R | R | R | R |
| Animal |  | UMA003 | S | S | S | R | R | R | R | R | R | R | R |
| Animal |  | UMA004 | S | S | S | R | R | R | R | R | R | R | R |
| Animal |  | UMA005 | S | S | S | R | R | R | R | R | R | R | R |
| Animal |  | UMA006 | S | S | S | R | R | R | R | R | R | R | R |
| Animal |  | UMA007 | S | S | S | R | R | R | R | R | R | R | R |
| Animal |  | UMA008 | S | S | S | R | R | R | R | R | R | R | R |
| Animal |  | UMA009 | S | S | S | R | R | R | R | R | R | R | R |
| Environmental |  | UME001 | S | S | S | R | R | R | R | R | R | R | R |
|  |  | | | | | | | | | | | | |
|  |  | *E. coli* ATCC 25922 | S | S | S | S | S | S | S | S | S | R | R |

Supplementary 2

Antibiotic susceptibility of strains used in the preliminary study (S- susceptible, R- resistant)

All strains are *B. pseudomallei* unless stated

|  | Antibiotic susceptibility (µg/ml) by disk diffusion | | | | | | | | | | |
| --- | --- | --- | --- | --- | --- | --- | --- | --- | --- | --- | --- |
| **Type of strains** |  | **CIP 5** | **C 10** | **DXT 30** | **IMI 10** | **MRP 10** | **TGC 15** | **SXT** | **CAZ 30** | **AUG--30** |  |
| Clinical | UMC002 | I | S | S | S | S | R | R | S | I |  |
| Clinical | UMC006 | I | R | S | S | S | R | R | S | I |  |
| Clinical | UMC007 |  |  |  |  |  |  |  |  |  | Not available |
| Clinical | UMC009 |  |  |  |  |  |  |  |  |  | Not available |
| Clinical | UMC010 | S | S | S | S | S | R | R | S | R |  |
| Clinical | UMC011 | I | S | S | S | S | R | R | S | S |  |
| Clinical | UMC012 |  |  |  |  |  |  |  |  |  | Not available |
| Clinical | UMC013 |  |  |  |  |  |  |  |  |  | Not available |
| Clinical | UMC014 | I | I | S | S | S | R | R | S | S |  |
| Clinical | UMC015 | I | I | S | S | S | R | R | S | I |  |
| Clinical | UMC016 | S | I | S | S | S | R | R | S | R |  |
| Clinical | UMC019 | S | S | S | S | S | R | R | S | R |  |
| Clinical | UMC020 | I | S | S | S | S | R | R | S | R |  |
| Clinical | UMC021 | S | S | S | S | S | R | R | S | I |  |
| Clinical | UMC022 | S | S | S | S | S | I | R | S | S |  |
| Clinical | UMC023 | R | R | S | S | S | R | R | S | R |  |
| Clinical | UMC024 | S | S | S | S | S | R | R | S | R |  |
| Clinical | UMC025 | S | S | S | S | S | R | R | S | R |  |
| Clinical | UMC026 | I | S | S | S | S | R | R | S | I |  |
| Clinical | UMC027 |  |  |  |  |  |  |  |  |  | Not available |
| Clinical | UMC028 |  |  |  |  |  |  |  |  |  | Not available |
| Clinical | UMC029 |  |  |  |  |  |  |  |  |  | Not available |
| Clinical | UMC030 |  |  |  |  |  |  |  |  |  | Not available |
| Clinical | UMC031 |  |  |  |  |  |  |  |  |  | Not available |
| Clinical | UMC032 (K96243) | S | I | S | S | S | R | R | S | I |  |
| Clinical | UMC033 | S | S | S | S | S | R | R | S | I |  |
| Clinical | UMC034 | S | S | S | S | S | R | R | S | S |  |
| Clinical | UMC035 | S | I | S | S | S | R | R | S | R |  |
| Clinical | UMC036 | I | I | S | S | S | R | R | S | I |  |
| Clinical | UMC038 |  |  |  |  |  |  |  |  |  | Not available |
| Clinical | UMC039 |  |  |  |  |  |  |  |  |  | Not available |
| Clinical | UMC040 | I | S | S | S | S | R | R | S | I |  |
| Clinical | UMC042 |  |  |  |  |  |  |  |  |  | Not available |
| Clinical | UMC043 |  |  |  |  |  |  |  |  |  | Not available |
| Clinical | UMC044 |  |  |  |  |  |  |  |  |  | Not available |
| Clinical | UMC045 |  |  |  |  |  |  |  |  |  | Not available |
| Clinical | UMC046 |  |  |  |  |  |  |  |  |  | Not available |
| Clinical | UMC047 | I | I | S | S | S | R | R | S | I |  |
| Clinical | UMC048 |  |  |  |  |  |  |  |  |  | Not available |
| Clinical | UMC049 | S | I | S | S | S | R | R | S | I |  |
| Clinical | UMC049L | S | I | S | S | S | R | R | S | I |  |
| Clinical | UMC049S | S | I | S | S | S | R | R | S | I |  |
| Clinical | UMC050 | I | S | S | S | S | R | R | S | R |  |
| Clinical | UMC051 |  |  |  |  |  |  |  |  |  | Not available |
| Clinical | UMC052 | S | S | S | S | S | R | R | S | S |  |
| Clinical | UMC053 | S | S | S | - | - | - | R | R | R |  |
| Clinical | UMC054A | I | I | S | S | S | R | I | S | I |  |
| Clinical | UMC054B | S | S | S | S | S | R | I | S | I |  |
| Clinical | UMC055 |  |  |  |  |  |  |  |  |  | Not available |
| Clinical | UMC056 | S | S | S | S | S | R | R | S | I |  |
| Clinical | UMC057 | I | S | S | S | S | R | R | I | R |  |
| Clinical | UMC058 | S | S | S | S | S | R | R | S | I |  |
| Clinical | UMC059 | S | I | S | S | S | R | I | S | I |  |
| Clinical | UMC061 | I | S | S | S | S | R | R | S | I |  |
| Clinical | UMC061A | I | S | S | S | S | R | R | S | I |  |
| Clinical | UMC061B | I | S | S | S | S | R | R | S | I |  |
| Clinical | UMC062 | I | S | S | S | S | R | R | S | I |  |
| Clinical | UMC063 | S | S | S | S | S | R | R | S | I |  |
| Clinical | UMC064 | I | I | S | S | S | R | I | S | R |  |
| Clinical | UMC065 | I | I | S | S | S | R | R | S | I |  |
| Clinical | UMC066 | S | I | S | S | S | R | I | S | S |  |
| Clinical | UMC067 | S | S | S | S | S | R | S | I | S |  |
| Clinical | UMC068 | S | S | S | S | S | R | R | S | S |  |
| Clinical | UMC069 | S | I | S | S | S | R | S | R | I |  |
| Clinical | UMC070 | I | I | S | S | S | R | I | S | S |  |
| Clinical | UMC071 | S | S | S | S | S | R | R | S | S |  |
| Clinical | UMC072 | S | S | S | S | S | R | R | S | S |  |
| Clinical | UMC073 | I | S | S | S | S | R | R | S | R |  |
| Clinical | UMC074 | I | I | S | S | S | R | I | S | I |  |
| Clinical | UMC074L | I | I | S | S | S | R | I | S | I |  |
| Clinical | UMC074S | I | I | S | S | S | R | I | S | I |  |
| Clinical | UMC076 |  |  |  |  |  |  |  |  |  | Not available |
| Clinical | UMC077 | I | I | S | S | S | R | S | S | I |  |
| Clinical | UMC078 |  |  |  |  |  |  |  |  |  | Not available |
| Clinical | UMC079 |  |  |  |  |  |  |  |  |  | Not available |
| Clinical | UMC080 |  |  |  |  |  |  |  |  |  | Not available |
| Clinical | UMC081 |  |  |  |  |  |  |  |  |  | Not available |
| Clinical | UMC082 |  |  |  |  |  |  |  |  |  | Not available |
| Clinical | UMC084 |  |  |  |  |  |  |  |  |  | Not available |
| Clinical | UMC085 |  |  |  |  |  |  |  |  |  | Not available |
| Clinical | UMC086 |  |  |  |  |  |  |  |  |  | Not available |
| Clinical | UMC087 |  |  |  |  |  |  |  |  |  | Not available |
| Clinical | UMC088 |  |  |  |  |  |  |  |  |  | Not available |
| Clinical | UMC089 |  |  |  |  |  |  |  |  |  | Not available |
| Clinical | UMC105 |  |  |  |  |  |  |  |  |  | Not available |
| Clinical | UMC106 |  |  |  |  |  |  |  |  |  | Not available |
| Clinical | UMC107 |  |  |  |  |  |  |  |  |  | Not available |
| Clinical | UMC108 |  |  |  |  |  |  |  |  |  | Not available |
| Clinical | UMC109 |  |  |  |  |  |  |  |  |  | Not available |
|  |  |  |  |  |  |  |  |  |  |  |  |
| Animal | UMA001 | S | I | S | S | S | S | I | S | S |  |
| Animal | UMA002 | I | S | S | S | S | I | I | S | R |  |
| Animal | UMA003 | S | S | S | S | S | S | I | S | R |  |
| Animal | UMA004 | S | S | S | S | S | S | S | S | S |  |
| Animal | UMA005 | I | S | S | S | S | I | S | S | S |  |
| Animal | UMA006 | S | S | S | S | S | S | S | S | I |  |
| Animal | UMA007 | I | S | S | S | S | R | R | R | R |  |
| Animal | UMA008 | I | S | S | S | S | S | S | S | S |  |
| Animal | UMA009 | I | S | S | S | S | I | S | S | S |  |
| Environmental | UME001 |  |  |  |  |  |  |  |  |  | Not available |
|  |  |  |  |  |  |  |  |  |  |  |  |
|  | E.coli ATCC 25922 | S | S | S | S | S | I | S | S | S |  |

Supplementary 3

Molar concentrations of antimicrobial agents used in this study

|  | Concentration in µM | | | | | | | | | | |
| --- | --- | --- | --- | --- | --- | --- | --- | --- | --- | --- | --- |
| Concentration of AMP (µg/ml) | TP1 | LL37 | Protegrin 1 | Magainin 2 | Sushi 1 | Sushi 3 | 1037 | 1018 | DJK5 | V2D | Ornithine |
| 1000 | 441.74 | 222.55 | 463.89 | 405.37 | 266.1 | 257.07 | Molecular weight was not revealed by collaborators    As such, molar concentration could not be calculated | | | | |
| 500 | 220.87 | 111.28 | 231.94 | - | - | - |  |  |  |  |  |
| 250 | 110.43 | 55.64 | 115.97 | - | - | - |  |  |  |  |  |
| 100 | 48.59 | 22.26 | 46.39 | - | - | - |  |  |  |  |  |
| 50 | 22.35 | 11.13 | 23.19 | - | - | - |  |  |  |  |  |
| 25 | 11.04 | 5.56 | 11.6 | - | - | - |  |  |  |  |  |
| 12.5 | 5.52 | 2.78 | 5.8 | - | - | - |  |  |  |  |  |
| 6.25 | 2.76 | 1.39 | 2.9 | - | - | - |  |  |  |  |  |
| 3.13 | 1.38 | 0.7 | 1.45 | - | - | - |  |  |  |  |  |
| 1.06 | 0.47 | 0.24 | 0.49 | - | - | - |  |  |  |  |  |
| 0 | 0 | 0 | 0 | - | - | - |  |  |  |  |  |

Relative Molecular Mass

| Antimicrobial agents |  | Molecular mass (g/mol) |
| --- | --- | --- |
| TP1 | : | 2263.78 |
| LL37 | : | 4493.30 |
| PG1 | : | 2155.70 |
| Magainin 2 | : | 2466.90 |
| Sushi 1 | : | 3758.00 |
| Sushi 3 | : | 3890.00 |
| Ceftazidime | : | 546.58 |
| Meropenem | : | 383.46 |

Formula

$Concentration in \mu M=(Concentration in (\mu g/mL) \times10^3)/(Relative Molecular Mass)$

Supplementary 4

Abbreviations from *E. coli* LPS (PDB ID: 1QFG) (Adapted from Ferguson et al. (2000) )

| No | Residue abbreviation | Name/ Formula/ InChl Key | 2D diagram |
| --- | --- | --- | --- |
| 1. | DAO | LAURIC ACID C_12_ H_24_ O_2_  POULHZVOKOAJMA-UHFFFAOYSA-N | 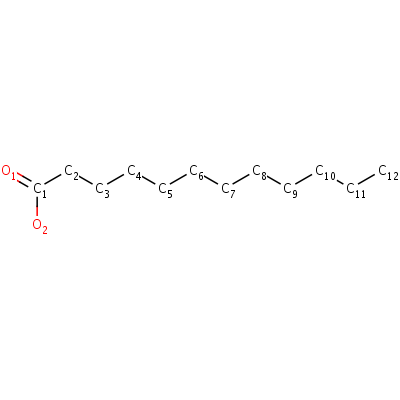 |
| 2. | DDQ | DECYLAMINE-N,N-DIMETHYL-N-OXIDE C_12_ H_27_ N O  ZRKZFNZPJKEWPC-UHFFFAOYSA-N | 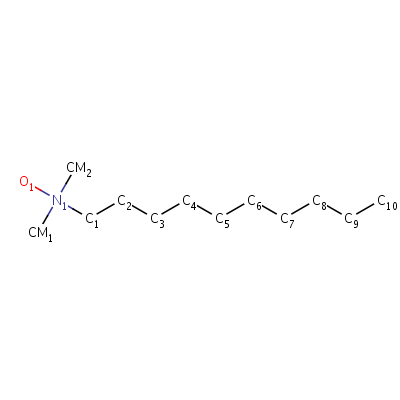 |
| 3. | DPO | DIPHOSPHATE O_7_ P_2_  XPPKVPWEQAFLFU-UHFFFAOYSA-J | 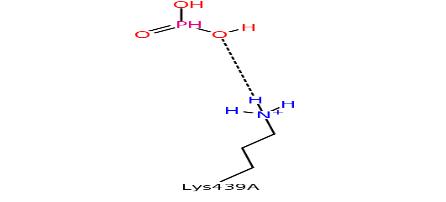 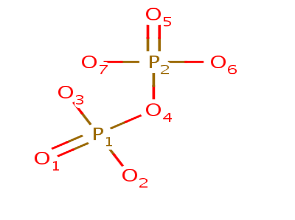 |
| 4. | FTT | 3-HYDROXY-TETRADECANOIC ACID 3-HYDROXY-MYRISTIC ACID (Synonym) C_14_ H_28_ O_3_  ATRNZOYKSNPPBF-CYBMUJFWSA-N | 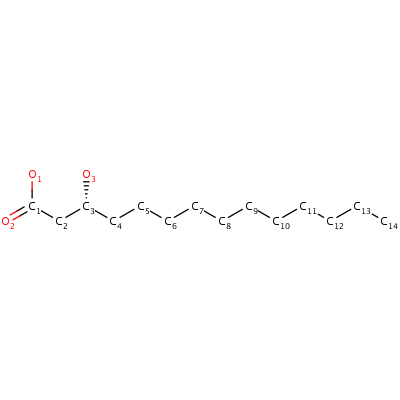 |
| 5. | GCN | 3-DEOXY-D-GLUCOSAMINE C_6_ H_13_ N O_4_  SNDZDGQLFKEBLF-MOJAZDJTSA-N | 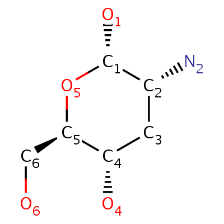 |
| 6. | GLA | ALPHA D-GALACTOSE C_6_ H_12_ O_6_  WQZGKKKJIJFFOK-PHYPRBDBSA-N | 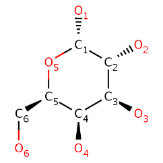 |
| 7. | GLC | ALPHA-D-GLUCOSE C_6_ H_12_ O_6_  WQZGKKKJIJFFOK-DVKNGEFBSA-N | 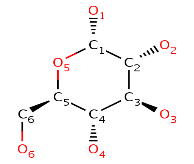 |
| 8. | GMH | L-GLYCERO-D-MANNO-HEPTOPYRANOSE C_7_ H_14_ O_7_  BGWQRWREUZVRGI-QQABCQGCSA-N | 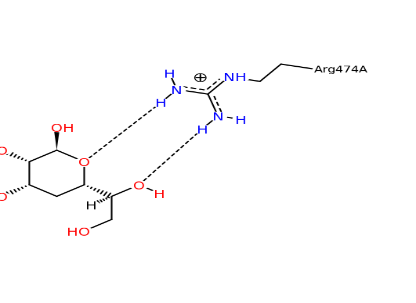 |
| 9. | GOL | GLYCEROL GLYCERIN; PROPANE-1,2,3-TRIOL (Synonym) C_3_ H_8_ O_3_  PEDCQBHIVMGVHV-UHFFFAOYSA-N | 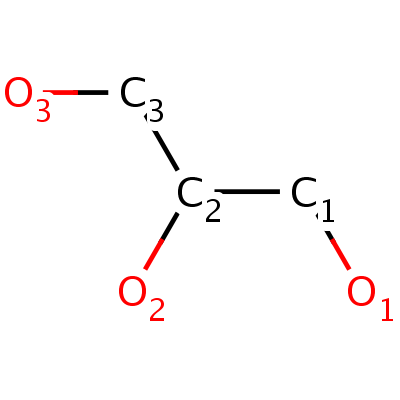 |
| 10. | KDO | 3-DEOXY-D-MANNO-OCT-2-ULOSONIC ACID C_8_ H_14_ O_8_  NNLZBVFSCVTSLA-HXUQBWEZSA-N | 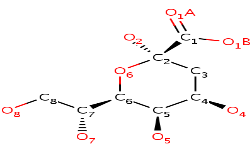 |
| 11. | MYR | MYRISTIC ACID C_14_ H_28_ O_2_  TUNFSRHWOTWDNC-UHFFFAOYSA-N | 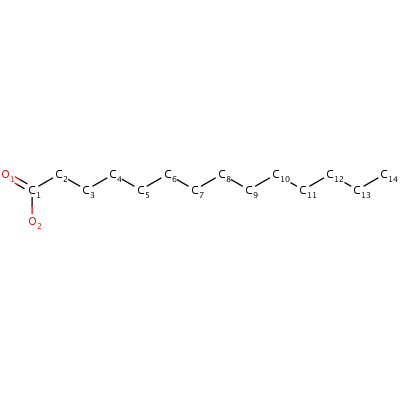 |
| 12. | PA1 | 2-amino-2-deoxy-alpha-D-glucopyranose C_6_ H_13_ N O_5_  MSWZFWKMSRAUBD-UKFBFLRUSA-N | 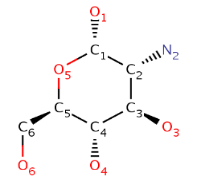 |
| 13. | PO4 | PHOSPHATE ION O_4_ P  NBIIXXVUZAFLBC-UHFFFAOYSA-K | 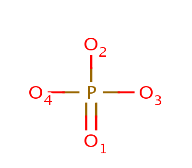 |
| 14. | NI | NICKEL (II) ION Ni  VEQPNABPJHWNSG-UHFFFAOYSA-N | 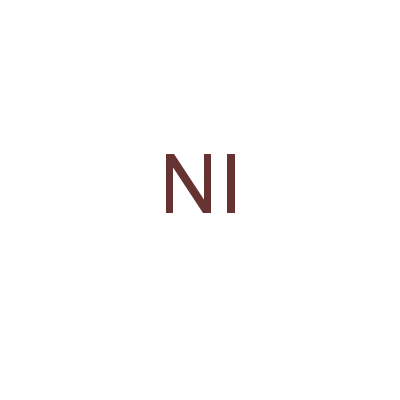 |

Supplementary 5

*B. pseudomallei* homology model verification

4.1 PROCHECK report


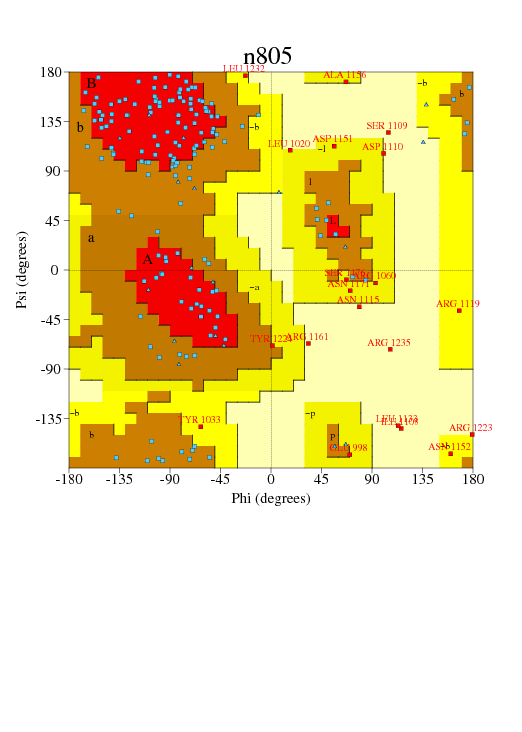


**4.1.1 Ramachandran Plot statistics**

**No. of**

**residues %-tage**

**------ ------**

Most favoured regions [A, B, L] 87 53.7%******

Additional allowed regions [a,b,l,p] 55 34.0%

Generously allowed regions [~a,~b,~l,~p] 12 7.4%

Disallowed regions [XX] 8 4.9%*****

---- ------

Non-glycine and non-proline residues 162 100.0%

End-residues (excl. Gly and Pro) 2

Glycine residues 19

Proline residues 12

----

Total number of residues 195

Based on an analysis of **118** structures of resolution of at least **2.0** Angstroms and *R*-factor no greater than **20.0** a good quality model would be expected to have over **90%** in the most favoured regions [A,B,L].

**4.1.2 G-Factors**

**Average**

**Parameter Score Score**

**--------- ----- -----**

Dihedral angles: -

Phi-psi distribution  **-1.63****

Chi1-chi2 distribution -0.91*

Chi1 only -0.49

Chi3 & chi4 0.58

Omega  **-1.62****

**-1.25****

=====

Main-chain covalent forces: -

Main-chain bond lengths -0.06

Main-chain bond angles  **-1.62****

-0.96*

=====

OVERALL AVERAGE  **-1.08****

=====

**G-factors** provide a measure of how **unusual**, or out-of-the-ordinary, a property is.

Values below -0.5* - unusual

Values below **-1.0**** - highly unusual

**Important note:** The main-chain bond-lengths and bond angles are compared with the Engh & Huber (1991) ideal values derived from small-molecule data. Therefore, structures refined using different restraints may show apparently large deviations from normality.

4.2 ERRAT report


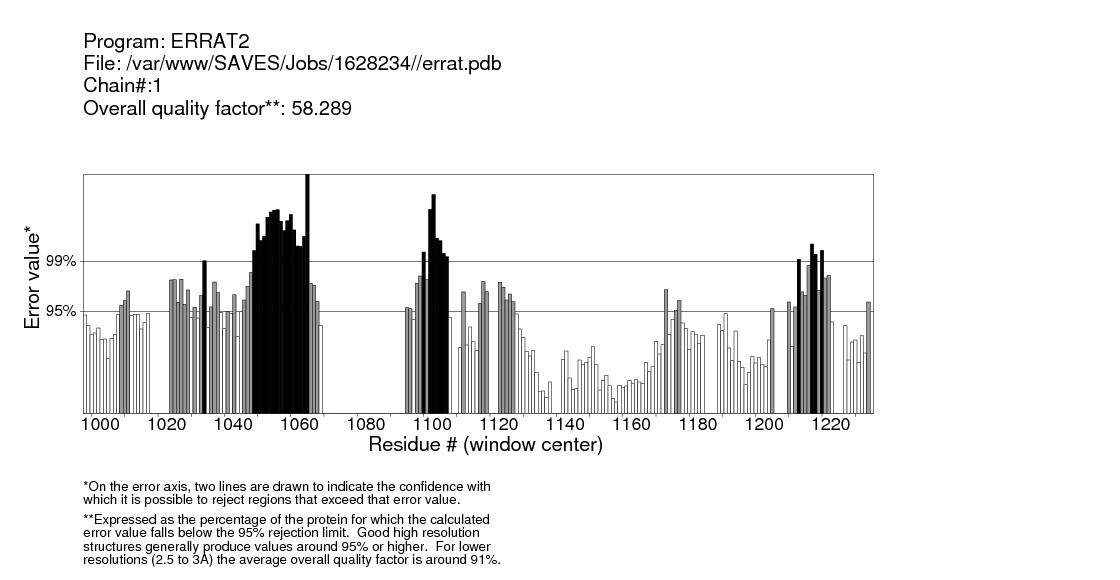


4.3 Verify 3D Results plot

76.41% of the residues had an averaged 3D-1D score >= 0.2


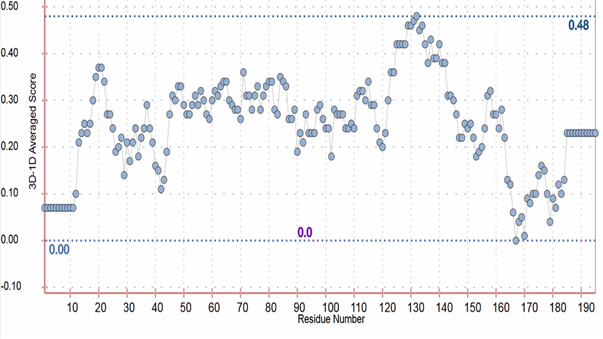


Supplementary 6

List of *B. pseudomallei* PDB protein molecules for molecular docking.

| **PDB ID** | ***Burkholderia pseudomallei* Molecules** |
| --- | --- |
| 2G0U | Solution structure of monomeric BSAL, the type III secretion needle protein |
| 2IXR | BipD |
| 2JOK | NMR structure of the catalytic domain of guanine nucleotide exchange factor BopE |
| 2MPE | Solution NMR structure for strain BPSl1050 |
| 2WLL | Potassium channel |
| 2X3Y | Crystal structure of GMHA |
| 2XBL | Crystal structure of GMHA in complex with product |
| 2Y78 | Crystal structure of BPSS1823, a Mip-like chaperone |
| 3E5Y | Crystal structure of Trmh family RNA methyltransferase |
| 3GQM | Crystal structure of cycle inhibiting factor |
| 3LAA | Crystal structure of the trimeric autotransporteradhesin head domain BpaA |
| 3LR3 | Periplasmic domain of the Riss sensor protein, low pH native structure |
| 3RQI | Crystal structure of a response regulator protein with a phosphorylated aspartic acid, calcium ion and citrate |
| 3S6O | Crystal structure of a polysaccharide deacetylase family protein |
| 3SLG | Crystal structure of Pbgp3 protein |
| 3TU8 | Crystal structure of the lethal factor 1 |
| 3UAM | Crystal structure of a chitin binding domain |
| 3ZS6 | The structural characterization of OppA |
| 4B5C | Crystal structure of the peptidoglycan-associated lipoprotein |
| 4C26 | NMR structure of the HicA toxin |
| 4CFI | 3D structure of FliC |
| 4F3P | Crystal structure of a glutamine-binding periplasmic protein in complex with glutamine |
| 4HCN | Crystal structure of protein CHBP in complex with ubiquitin |
| 4HCP | Crystal structure of effector protein CHBP in complex with nedd8 |
| 4UT1 | The structure of the flagellar hook junction protein FlgK |
| 4WNY | Crystal structure of a protein from the universal stress protein family |

Supplementary 7

Data of residue interactions of TP1 in binding with amino acid acids of potential target proteins (interaction within 3Å of peptide are highlighted in bold number, interaction energy values less than -20 kcal/mol are shaded)

| **TP1 Amino Acid residue** | 2G0U  (1) | 2IXR  (2) | 2JOK  (3) | 2MPE  (4) | 2WLL  (5) | 2X3Y  (6) | 2XBL  (7) | 2Y78  (8) | 3E5Y  (9) | 3GQM  (10) | 3LAA  (11) | 3LR3  (12) | 3QRI  (13) | 3S6O  (14) |
| --- | --- | --- | --- | --- | --- | --- | --- | --- | --- | --- | --- | --- | --- | --- |
| LYS1 | **-68.29** | **-36.13** | **44.37** | **-21.72** | **25.25** | **25.08** | **-60.30** | **-2.48** | **-27.26** | **13.23** | **-13.89** | **-24.45** | **-56.20** | **-23.51** |
| TRP2 | **4.23** | **-10.00** | **9.71** | **1.89** | **-16.72** | **9.16** | **2.48** | **-7.32** | **15.44** | **-7.48** | **-20.29** | 5.39 | **22.84** | **-9.28** |
| CYS3 | 11.32 | 2.86 | **-12.24** | 0.26 | **40.14** | **-14.31** | **-6.77** | 5.47 | -6.96 | 9.72 | **-5.36** | 2.99 | **-28.23** | **23.05** |
| PHE4 | -0.04 | -8.11 | -0.27 | **-4.10** | **-2.82** | 2.63 | **-16.43** | **9.58** | 0.12 | **5.18** | **923.08** | -2.05 | **4.13** | 10.37 |
| ARG5 | -5.70 | **-41.03** | **-4.29** | -33.24 | **-12.42** | **67.03** | **-8.91** | **-40.74** | 17.93 | 2.89 | **-38.40** | **17.99** | **26.39** | **11.69** |
| VAL6 | -3.33 | **29.30** | 5.07 | **14.97** | **4.29** | **15.73** | **-8.42** | 11.73 | 15.16 | 4.21 | -10.01 | **13.07** | **17.84** | -2.48 |
| CYS7 | 9.78 | **-5.10** | 1.10 | -7.72 | **-6.64** | -14.68 | **-20.90** | **-16.32** | -2.50 | 2.33 | **1.12** | **6.94** | **-2.97** | **4.96** |
| TYR8 | 7.45 | **-8.96** | 4.91 | **-2.83** | **-12.85** | -10.14 | -6.95 | 7.19 | -3.04 | **-6.23** | **-4.33** | **15.31** | **-7.03** | -24.14 |
| ARG9 | 39.84 | **-2.38** | -9.17 | 8.94 | **22.54** | **-68.17** | 23.19 | -30.70 | -0.97 | **0.06** | 3.77 | -0.96 | 59.13 | **-53.29** |
| GLY10 | -0.97 | 14.76 | **15.47** | **2.78** | -3.46 | -1.36 | -4.58 | -5.23 | -6.65 | **2.43** | -6.73 | 6.56 | 5.30 | 5.98 |
| ILE11 | 3.83 | **-21.77** | **-18.72** | **0.80** | 2.74 | -4.89 | 0.49 | -2.00 | 1.79 | -3.90 | -10.98 | **-20.55** | -22.33 | -0.33 |
| CYS12 | **9.62** | -1.14 | **-1.98** | -4.37 | 4.62 | 7.01 | **-4.03** | -2.42 | 4.20 | 5.50 | **-7.62** | -5.95 | -1.56 | **-6.65** |
| TYR13 | **11.89** | 10.27 | **143.93** | **-1.37** | 0.24 | -12.60 | 3.84 | 1.22 | **5.72** | **-17.86** | 0.92 | **-11.12** | -0.02 | -8.56 |
| ARG14 | **-37.34** | -59.79 | **-16.96** | -3.76 | 47.98 | -34.51 | **47.86** | -15.68 | **6.44** | -53.42 | **17.68** | 21.53 | 2.97 | **0.10** |
| ARG15 | **-22.23** | 4.22 | **52.26** | **-1.23** | 7.21 | **115.94** | -1.97 | 0.95 | **-9.52** | **-19.86** | 8.19 | **-35.44** | **3.75** | **13.99** |
| CYS16 | **5.68** | -2.28 | **-11.42** | 1.02 | **-13.90** | **-10.11** | 0.75 | -3.22 | **-3.94** | -12.98 | **7.43** | 3.76 | 13.43 | **-13.08** |
| ARG17 | **-28.05** | **8.71** | **34.94** | **-35.76** | 6.58 | **-0.65** | **-40.77** | 6.67 | **192.41** | **-154.58** | **-23.86** | **-82.36** | **13.38** | **-20.52** |

Data of residue interactions of TP1 in binding with amino acid acids of potential target proteins (interaction within 3Å of peptide are highlighted in bold number, interaction energy values less than -20 kcal/mol are shaded) (continued)

| **TP1 Amino Acid residue** | 3SLG  (15) | 3TU8  (16) | 3UAM  (17) | 3ZS6  (18) | 4B5C  (19) | 4C26  (20) | 4CFI  (21) | 4F3P  (22) | 4HCN  (23) | 4HCP  (24) | 4UT1  (25) | 4WNY  (26) | Sum of interaction energy | Number of values less than -20 kcal/mol |
| --- | --- | --- | --- | --- | --- | --- | --- | --- | --- | --- | --- | --- | --- | --- |
| LYS1 | 10.69 | **-47.92** | **6.63** | **25.88** | **36.49** | **30.62** | **-36.28** | **-6.77** | **-48.51** | **-57.26** | **-1.81** | **-22.03** | -256.93 | 12 |
| TRP2 | 8.65 | **-5.91** | **154.18** | **-16.06** | **-2.21** | 6.28 | -5.04 | **-15.98** | **-13.46** | **-6.69** | **8.50** | **-15.21** | 156.98 | 1 |
| CYS3 | 0.38 | **-8.18** | 0.68 | 4.73 | -1.29 | -0.80 | -5.10 | -4.24 | 5.50 | 3.74 | **-9.60** | 2.15 | 14.82 | 1 |
| PHE4 | **0.32** | -18.24 | -16.22 | 5.42 | 8.20 | **-0.92** | **-17.04** | -0.87 | 18.75 | -2.52 | **5.10** | **2.94** | 887.13 | 0 |
| ARG5 | **-11.43** | **-51.39** | **-23.43** | -36.16 | **-28.65** | **10.35** | **1.94** | **-51.95** | -4.57 | 8.69 | **-8.53** | **0.54** | -127.08 | 9 |
| VAL6 | **0.35** | **1.33** | 9.37 | 10.85 | -7.56 | **19.99** | **73.41** | -3.68 | -6.40 | -13.11 | -2.16 | -2.57 | 118.19 | 0 |
| CYS7 | -11.21 | **-11.42** | **-0.77** | 3.64 | -1.66 | -1.58 | **-18.51** | 11.17 | 1.02 | 5.05 | -3.25 | 1.63 | -74.01 | 1 |
| TYR8 | **-23.41** | 3.33 | **-19.08** | -2.34 | -8.31 | **-0.99** | **3.53** | -2.59 | -5.24 | -18.17 | 6.25 | **-13.38** | -90.81 | 2 |
| ARG9 | -22.07 | **-34.99** | **-84.18** | -12.21 | 0.93 | 44.53 | **-49.09** | 44.68 | **-70.60** | -0.10 | 4.28 | 6.35 | -149.41 | 8 |
| GLY10 | **4.32** | 1.03 | **-16.58** | **24.51** | -13.88 | 9.69 | -5.24 | **-7.29** | **-1.25** | -9.00 | **-7.13** | -2.26 | 13.07 | 0 |
| ILE11 | 3.60 | -4.90 | 11.57 | **4.27** | 10.14 | **8.54** | 5.24 | -6.91 | 2.73 | **-2.63** | **8.14** | -6.43 | -85.53 | 3 |
| CYS12 | **14.73** | 7.23 | 36.93 | **21.17** | **-13.55** | -2.28 | 7.32 | **2.41** | **11.79** | -2.67 | **-2.09** | -6.51 | 54.13 | 0 |
| TYR13 | **3.05** | -3.98 | -13.30 | **-17.08** | **4.59** | **-9.16** | -12.18 | 8.89 | **12.68** | **149.59** | 7.52 | **13.22** | 112.27 | 0 |
| ARG14 | **-69.12** | **72.58** | **-0.73** | **-26.31** | **9.30** | 9.63 | **-39.23** | **-12.04** | **40.77** | **23.38** | **-12.18** | -10.11 | -74.19 | 7 |
| ARG15 | **5.66** | -5.17 | **34.42** | **52.77** | **-9.15** | **70.09** | -1.12 | **-19.68** | **-45.00** | **-21.45** | 2.05 | **-63.41** | 151.18 | 5 |
| CYS16 | -7.42 | **5.08** | -10.10 | **-27.34** | **3.15** | -2.34 | -7.20 | **17.29** | -2.02 | -6.25 | **5.10** | -7.00 | -51.27 | 1 |
| ARG17 | -34.90 | **-6.43** | **5.78** | **-45.50** | **-30.79** | **57.99** | **17.72** | **23.32** | **-5.52** | **-11.23** | **-18.29** | **17.26** | -159.42 | 10 |

Supplementary 8

Seven TP1-*B. pseudomallei* protein complexes based on interaction energy from peptide less than -100 kcal/mol with the detail for hydrogen bonding and the Pi interactions.

| **PDB ID** | **Total**  **Interaction Energy**  **(kcal/mol)**  **(A+B)** | **VDW Energy**  **(kcal/mol)**  **(A)** | **Electrostatic energy**  **(kcal/mol)**  **(B)** | **Binding Affinity**  **(kcal/mol)** | **Hydrogen Bonds** | **Hydrogen bond details** | **Intermolecular Pi Interactions**  **(C-π) Cation-Pi Interactions**  **(π-π) Pi- Pi Interaction**  **(π-σ) Pi-Sigma Interactions** |
| --- | --- | --- | --- | --- | --- | --- | --- |
| 3GQM  (Cycle inhibiting factor) | -230.75 | -8.10 | -222.65 | -8.5(-8.0) | 5 | TP1:TYR13:HH - 3GQM:SER243:O  TP1:ARG15:HH21 - 3GQM:SER112:OG  TP1:ARG17:HE - 3GQM:GLU114:OE1  TP1:ARG17:HE - 3GQM:GLU114:OE2  TP1:ARG17:HH12 - 3GQM:GLU114:OE1 | (C-π) TP1:TYR8 - 3GQM:LYS132:NZ  TP1:TRP2 - 3GQM:LYS201:NZ  TP1:TRP2 - 3GQM:LYS201:NZ |
| 3SLG  (Pbgp 3 protein) | -127.80 | -53.17 | -74.63 | -10.9(-9.9) | 5 | TP1:LYS1:HZ2 - 3SLG:SER107:OG  TP1:LYS1:HZ3 - 3SLG:PRO103:O  TP1:ARG9:HH22 - 3SLG:LYS110:O  TP1:ARG14:HE - 3SLG:GLU99:OE2  3SLG:LYS110:HZ1 - TP1:ARG17:O | (C-π) TP1:TYR8 - 3SLG:LYS110:NZ  TP1:TYR13 - 3SLG:LYS61:NZ  TP1:PHE4 - 3SLG:LYS61:NZ |
| 2IXR  (Bip D) | -126.57 | -48.63 | -77.94 | -8.0(-7.4) | 3 | TP1:ARG5:HE-2IXR:SER280:OG  TP1:CYS7:HN-2IXR:GLU277:HO  TP1:ARG9:HH11-2IXR:LYS214:O | (C-π) TP1:TRP2 - 2IXR:ARG48:NE  TP1:TRP2 - 2IXR:ARG48:NE  (π-σ)2IXR:HIS281 - TP1:TYR8:HB1 |
| 4HCN  (Effector protein) | -109.35 | -9.28 | -100.07 | -7.8(-6.8) | 4 | TP1:LYS1:HZ3 - 4HCN:ASP118:OD1  TP1:LYS1:HZ3 - 4HCN:ASP118:OD2  TP1:CYS12:HN - 4HCN:THR12:O  4HCN:THR14:HN - TP1:GLY10:O | (C-π) TP1:TYR13 - 4HCN:ARG109:NE  TP1:TYR13 - 4HCN:LYS11:NZ |
| 3TU8  (Lethal factor 1) | -107.93 | 23.84 | -131.77 | -6.7(-5.8) | 2 | TP1:ARG5:HH22 - 3TU8:SER185:OG  TP1:ARG17:HH21 - 3TU8:ALA198:O | (C-π) TP1:TRP2 - 3TU8:LYS14:NZ  TP1:TRP2 - 3TU8:LYS14:NZ |
| 4WNY  (Universal stress protein) | -104.83 | -41.73 | -63.10 | -7.4 (-6.4) | 4 | TP1:LYS1:HT2 - 4WNY:SER12:OG  TP1:TRP2:HE1 - 4WNY:TYR37:OH  TP1:ARG15:HH11 - 4WNY:ASP40:OD2  TP1:NH218:HN2 - 4WNY:ASP10:OD2 | - |
| 2XBL  (GMHA) | -101.42 | -51.48 | -49.94 | -8.2(-7.6) | 6 | TP1:LYS1:HT1-2XBL:GLU115:OE1  TP1:LYS1:HT2-2XBL:GLU115:OE2  TP1:LYS1:HT3-2XBL:ALA140:O  TP1:TRP2:HN-2XBL:ALA140:O  TP1:ARG5:HH22-2XBL:ALA111:O  TP1:NH218:HN1-2XBL:GLU137:OE1 | - |
